# Cross-Cultural Adaptation and Initial Psychometric Evaluation of the Kazakh-Language Sino-Nasal Outcome Test-22 (SNOT-22) in Patients with Chronic Rhinosinusitis with Nasal Polyps

**DOI:** 10.3390/jcm15197384

**Published:** 2026-09-23

**Authors:** Bayan Aigozhina, Rais Tulebaeyv, Gulmira Mukhamadiyeva, Talapbek Azhenov, Sayazhan Stanova, Gulzira Shagyrbayeva, Mukadas Umarova, Kurmanay Soltanbayeva, Zhanetta Mukanova, Radda Mesheleva-Bekh, Jerome R. Lechien, Kalamkas Sagandykova

**Affiliations:** 1 Department of Otorhinolaryngology, Astana Medical University, Astana 010000, Kazakhstan; aigozhina.b@amu.kz (B.A.); tulebaev.r@amu.kz (R.T.); mukhamadieva.g@amu.kz (G.M.); mukanova.zh@amu.kz (Z.M.); mescheleva.r@amu.kz (R.M.-B.); 2 City Clinical Hospital No. 5, Almaty 050051, Kazakhstan; aysafistar@gmail.com (S.S.); s.kurmanay-80@mail.ru (K.S.); 3 Ermen Group Limited Liability Company, Astana 010000, Kazakhstan; gulzirashagyrbayeva@gmail.com; 4 «M Clinica» Limited Liability Company, Astana 010000, Kazakhstan; dr.mukadas.umarova@gmail.com; 5 Department of General Surgery, Faculty of Medicine and Pharmacy, University of Mons (UMONS), B7000 Mons, Belgium; jerome.lechien@umons.ac.be; 6 “University Medical Center” Corporate Fund, School of Medicine, Astana 010000, Kazakhstan

**Keywords:** chronic rhinosinusitis with nasal polyps, SNOT-22, patient-reported outcome measures, quality of life, cross-cultural adaptation, psychometric evaluation, Kazakh language

## Abstract

**Background/Objectives:** Chronic rhinosinusitis with nasal polyps (CRSwNP) is associated with substantial impairment of the health-related quality of life. The Sino-Nasal Outcome Test-22 (SNOT-22) is one of the most widely used patient-reported outcome measures in chronic rhinosinusitis. To our knowledge, no published psychometric evaluation of a Kazakh-language SNOT-22 has previously been reported. The aim of this study was to perform the cross-cultural adaptation and evaluate selected measurement properties of the Kazakh-language SNOT-22 in adults with CRSwNP. **Methods:** This prospective single-center study included 151 participants: 101 patients with CRSwNP (vitamin D supplementation group, *n* = 50; standard-treatment group, *n* = 51) and 50 healthy volunteers. Both patient groups underwent endoscopic sinus surgery and received topical glucocorticosteroids; adjunctive vitamin D was administered only in the vitamin D supplementation group according to the prespecified protocol. Cross-cultural adaptation was conducted according to ISPOR recommendations. The selected measurement properties examined were internal consistency, known-group validity, responsiveness, and exploratory associations with Lund–Kennedy endoscopic scores. Test–retest reliability and structural validity were not assessed. **Results**: Baseline SNOT-22 scores were substantially higher in patients with CRSwNP than in healthy volunteers (*p* < 0.001), although the interpretation of this known-group comparison is limited by significant age and sex imbalance between groups. In the combined target CRSwNP sample with complete item-level data (*n* = 99), Cronbach’s alpha was 0.921. Subgroup coefficients were 0.901 in the vitamin D supplementation group and 0.934 in the standard-treatment group, while the coefficient in healthy volunteers was 0.891. After one month, SNOT-22 scores decreased substantially in both patient groups (both *p* < 0.001), supporting the instrument’s ability to detect change over time. Baseline correlations with Lund–Kennedy scores were very weak and non-significant; stronger correlations were observed only after treatment. **Conclusions:** The Kazakh-language SNOT-22 showed promising initial measurement properties, including high internal consistency and the ability to distinguish patients from healthy volunteers and to detect longitudinal score changes. However, the psychometric evaluation remains incomplete because test–retest reliability, measurement error, and structural validity were not assessed, and evidence for convergent validity was limited. Further validation in larger, demographically balanced samples is required.

## 1. Introduction

Chronic rhinosinusitis (CRS) is a heterogeneous inflammatory disease of the nasal cavity and paranasal sinuses that affects approximately 5–12% of the adult population worldwide and imposes a substantial clinical and socioeconomic burden [1,2]. Chronic rhinosinusitis with nasal polyps (CRSwNP), one of the major clinical phenotypes of the CRS, is characterized by persistent sinonasal symptoms, impaired olfaction, recurrent disease, and a frequent need for prolonged medical treatment or endoscopic sinus surgery [1,2]. According to the European Position Paper on Rhinosinusitis and Nasal Polyps (EPOS 2020), severe CRSwNP is predominantly associated with type 2 inflammation. This inflammatory endotype is characterized by interleukin (IL)-4, IL-5, and IL-13 signaling, eosinophilic infiltration, and increased local immunoglobulin E production, contributing to persistent inflammation, disease recurrence, and impaired health-related quality of life (HRQoL) [1,2,3,4]. Histopathological and ultrastructural studies of CRSwNP mucosa have additionally demonstrated prominent stromal edema, eosinophil-rich inflammatory infiltrates, mucous-cell hyperplasia, biofilm-associated changes, and increased local IL-33, supporting the importance of epithelial and local inflammatory mechanisms in the disease [5].

Over the past decade, patient-reported outcome measures (PROMs) have become an integral component of the clinical assessment of patients with CRS. Unlike endoscopic or radiological findings, PROMs directly capture patients’ perceptions of symptom burden, functional impairment, and treatment outcomes, thereby complementing objective clinical measures and supporting patient-centered care. Among disease-specific PROMs, the Sino-Nasal Outcome Test-22 (SNOT-22) is one of the most widely used instruments for assessing HRQoL in patients with chronic rhinosinusitis. Its widespread use is supported by established reliability, responsiveness, clinical interpretability, and applicability in both clinical practice and research [6,7,8,9,10].

The SNOT-22 has been translated and validated in numerous linguistic and cultural settings, including Arabic, Thai, Bahasa Malaysia, Polish, Hindi, Persian, Italian, Danish, and Brazilian Portuguese populations [7,11,12,13,14,15,16,17,18,19]. Although these studies have consistently demonstrated favorable measurement properties, translated PROMs cannot be assumed to perform equivalently across different linguistic and cultural settings. Variations in language structure, cultural interpretation of symptoms, and healthcare context may influence questionnaire performance and measurement characteristics. Therefore, international methodological guidelines, including those developed by the International Society for Pharmacoeconomics and Outcomes Research (ISPOR) and the COnsensus-based Standards for the selection of health Measurement INstruments (COSMIN), recommend rigorous cross-cultural adaptation followed by the evaluation of relevant measurement properties before a translated PROM is implemented in clinical practice or research [20,21,22,23,24,25].

Kazakh is the official state language of Kazakhstan and is widely used in everyday communication and healthcare settings. To our knowledge, no published psychometric evaluation of a Kazakh-language version of the SNOT-22 has previously been reported. Given the established role of validated disease-specific PROMs in the assessment of chronic rhinosinusitis, a culturally and linguistically adapted Kazakh-language instrument is needed to support standardized evaluation of patient-reported outcomes in Kazakh-speaking patients [6,7,10,26].

Therefore, the present study aimed to perform the cross-cultural adaptation of the Kazakh-language SNOT-22 and evaluate selected measurement properties in adults with chronic rhinosinusitis with nasal polyps.

## 2. Materials and Methods

### 2.1. Study Design and Participants

This prospective single-center comparative study was conducted between 2025 and 2026 at City Polyclinic No. 8, Astana, Kazakhstan. The study protocol was approved by the Local Bioethics Committee of Astana Medical University (Approval No. 8, 25 May 2025), and written informed consent, including consent for publication, was obtained from all participants before enrollment.

A total of 151 participants were enrolled. The study population comprised 101 consecutive adults with chronic rhinosinusitis with nasal polyps (CRSwNP) diagnosed according to the European Position Paper on Rhinosinusitis and Nasal Polyps (EPOS 2020) criteria and 50 healthy volunteers without a history of chronic sinonasal disease [1]. Healthy volunteers were included to evaluate the known-group validity of the questionnaire.

Patients with CRSwNP were allocated according to the therapeutic strategy used in the parent prospective clinical study to a vitamin D supplementation group (*n* = 50) and a standard treatment group (*n* = 51). Both groups underwent endoscopic sinus surgery and received topical glucocorticosteroids as standard background treatment. Serum 25-hydroxyvitamin D [25(OH)D] concentrations were measured in both groups. In the vitamin D supplementation group, patients with baseline serum 25(OH)D concentrations <30 ng/mL received adjunctive vitamin D at a dose of 10,000 IU/day for 3 months. Serum 25(OH)D was reassessed after 3 months; when the target concentration had been achieved, patients were switched to a maintenance dose of 2000 IU/day for an additional 3 months. The standard treatment group received the same surgical and topical glucocorticosteroid treatment without adjunctive vitamin D supplementation. Nasal polyp recurrence was assessed during the 6-month postoperative follow-up in the parent clinical study. Treatment efficacy was not an outcome of the present psychometric evaluation; the two clinical cohorts were used only for within-group assessment of longitudinal change in SNOT-22 scores.

To evaluate responsiveness, the SNOT-22 questionnaire was administered at baseline and repeated one month after treatment. This 1-month assessment was distinct from the 3-month reassessment of serum 25(OH)D and the 6-month postoperative assessment of nasal polyp recurrence. No between-group comparison of treatment effectiveness was used to establish questionnaire validity; repeated SNOT-22 measurements were analyzed primarily to determine whether the instrument detected change over time. Test–retest reliability was not assessed because the protocol did not include a second administration in clinically stable participants before treatment. The one-month patient reassessment occurred after surgery and medical therapy, when genuine clinical change was expected, and was therefore unsuitable for estimating score stability. Healthy volunteers completed the questionnaire once and were included specifically for assessment of known-groups validity.

### 2.2. Eligibility Criteria

Patients were eligible if they were aged ≥18 years, had CRSwNP diagnosed according to EPOS 2020 criteria [1], were native speakers or fluent users of the Kazakh language, and were able to complete the questionnaire independently. Patients were excluded if they had sinonasal malignancy, granulomatous or systemic inflammatory diseases affecting the upper airways, cognitive impairment preventing reliable questionnaire completion, or were unable to read or understand written Kazakh. Healthy volunteers met the same language eligibility criteria and were required to have no history or current symptoms of chronic rhinosinusitis or other significant otorhinolaryngological disease, no sinonasal symptoms during the preceding 3 months, and a normal nasal endoscopic examination at screening. Individuals with an acute upper respiratory tract infection at enrollment or within the preceding 4 weeks, exacerbated allergic rhinitis, or use of topical or systemic glucocorticosteroids during the preceding 6 months were not eligible as healthy volunteers. Healthy volunteer status was therefore defined clinically rather than by a SNOT-22 score cutoff, because the questionnaire also contains sleep-related, fatigue, functional, and emotional items that are not specific to chronic rhinosinusitis. Healthy volunteers served as the reference group for assessment of known-groups validity.

### 2.3. Cross-Cultural Adaptation

The Kazakh-language version of the Sino-Nasal Outcome Test-22 (SNOT-22) was developed following internationally accepted recommendations for the cross-cultural adaptation of patient-reported outcome measures proposed by the International Society for Pharmacoeconomics and Outcomes Research (ISPOR) and Beaton et al. [20,21].

The adaptation process comprised five sequential stages. First, two independent bilingual translators, whose native language was Kazakh, produced separate forward translations of the original English questionnaire. The authorized Russian-language adaptation was available as a linguistic reference during reconciliation and cultural adaptation. Second, the translated versions were compared and reconciled into a single consensus version by the research team. Third, the reconciled Kazakh version was independently back-translated into English by two bilingual translators who were blinded to the original questionnaire. Fourth, all versions were reviewed by a multidisciplinary expert committee consisting of otorhinolaryngologists, a professional translator, and a linguist to evaluate semantic, idiomatic, experiential, and conceptual equivalence and to produce the pre-final version. Finally, cognitive debriefing was conducted in ten adult patients with CRSwNP to assess the clarity, comprehensibility, cultural relevance, and acceptability of each questionnaire item.

No substantial linguistic, semantic, or cultural issues requiring modification of the questionnaire were identified during cognitive debriefing. The pre-final version was therefore accepted as the final Kazakh-language version of the SNOT-22 for subsequent evaluation of its measurement properties.

Written permission to use the SNOT-22 and the Kazakh-language translation in the present non-commercial academic study was obtained from Washington University in St. Louis, the official copyright holder and licensing authority for the instrument. Documentation of the permission is available upon request.

### 2.4. Clinical Assessment

Disease-specific health-related quality of life (HRQoL) was assessed using the Kazakh-language version of the Sino-Nasal Outcome Test-22 (SNOT-22), a disease-specific patient-reported outcome measure (PROM) for chronic rhinosinusitis. The questionnaire comprises 22 individual items. Each item is scored on a six-point Likert scale ranging from 0 (“no problem”) to 5 (“problem as bad as it can be”), yielding a total score from 0 to 110, with higher scores indicating greater patient-reported disease burden [7,10,27,28]. No domain-level scoring or factor structure was assumed in the present study. The structural layout and scoring system of the instrument are schematically illustrated in Figure 1 below.

Objective endoscopic disease severity was assessed using the Lund-Kennedy endoscopic scoring system [29].

Clinical assessment was performed at baseline before initiation of treatment and repeated one month after treatment initiation. The follow-up assessment was conducted solely to evaluate responsiveness of the questionnaire and not to compare treatment effectiveness between patient groups.

### 2.5. Assessment of Selected Measurement Properties

The measurement properties of the Kazakh-language SNOT-22 that were evaluated in the present study were selected in accordance with the COSMIN framework for studies of patient-reported outcome measures [22,23,24,25].

### 2.6. Internal Consistency

Internal consistency was evaluated using Cronbach’s alpha coefficient. The primary estimate was calculated in the combined CRSwNP patient sample because patients with CRSwNP constitute the target population of the instrument in the present study. Additional subgroup estimates were calculated separately for the vitamin D supplementation group, the standard treatment group, and healthy volunteers; the pooled all-participant estimate was retained only as a secondary descriptive analysis.

Corrected item-total correlations were calculated for each questionnaire item.

The influence of individual items on the overall Cronbach’s alpha coefficient was examined using Cronbach’s alpha if item deleted.

Cronbach’s alpha values ≥ 0.70 were considered acceptable; higher coefficients were interpreted cautiously in the context of structural validity [22,23,24].

### 2.7. Known-Group Validity

Known-group validity was assessed by comparing SNOT-22 total scores between patients with CRSwNP and healthy volunteers. Because questionnaire scores were not normally distributed, comparisons were performed using non-parametric statistical methods.

### 2.8. Exploratory Association with Endoscopic Findings

As exploratory evidence related to construct validity, associations between SNOT-22 total scores and Lund–Kennedy endoscopic scores were examined using Spearman’s rank correlation coefficient. Because SNOT-22 and Lund–Kennedy assess different aspects of CRSwNP and no a priori correlation hypothesis or independent patient-reported comparator was specified, these analyses were not considered sufficient on their own to establish convergent validity. Correlations were examined separately at baseline and after one month.

### 2.9. Responsiveness

Responsiveness was assessed by comparing baseline and one-month SNOT-22 scores within each clinical group. Changes over time were analyzed using the Wilcoxon signed-rank test. Cohen’s d_z_ was retained as a standardized descriptive index of change magnitude; statistical significance and effect size were not interpreted as evidence of treatment efficacy or as proof of clinically important change. No minimal clinically important difference (MCID) or patient-reported external anchor was evaluated in this study.

### 2.10. Sample Size Considerations

No separate a priori sample size calculation was performed specifically for the psychometric analyses because the study used the participant cohort of the parent prospective clinical study. The achieved sample was therefore evaluated retrospectively against COSMIN methodological guidance. Complete item-level data were available for 149 participants overall and 99 patients with CRSwNP. For hypothesis testing approaches to construct validity and responsiveness, COSMIN guidance generally regards samples of 50–99 participants as adequate and samples of at least 100 as very good, while interpretation must also consider the specific analysis and target population [30]. The two clinical subgroups each contained approximately 50 participants and therefore permitted the planned within-group and exploratory analyses, although subgroup estimates remain less precise than estimates from larger samples. Sample size alone does not compensate for the absence of structural validity assessment; the study was not designed for factor-analytic evaluation of the 22-item instrument or for measurement invariance analyses, which require a larger independent patient sample.

### 2.11. Statistical Analysis

Statistical analyses were performed using IBM SPSS Statistics version 20.0 (IBM Corp., Armonk, NY, USA). Continuous variables are presented as the mean ± standard deviation (SD) and, for non-normally distributed questionnaire and endoscopic scores, additionally as median and interquartile range (IQR). Categorical variables are presented as frequencies and percentages.

The distribution of continuous variables was assessed using the Shapiro–Wilk test together with visual inspection of histograms and Q–Q plots. Because most questionnaire scores were not normally distributed, non-parametric statistical methods were applied throughout the study.

Comparisons among the three independent groups were performed using the Kruskal–Wallis test for continuous variables and Pearson’s x^2^ test for categorical variables. Baseline Lund–Kennedy endoscopic scores were compared between the two patient groups using the Mann–Whitney U test.

Within-group changes between baseline and the one-month follow-up were analyzed using the Wilcoxon signed-rank test. The magnitude of longitudinal change was quantified using Cohen’s d_z_, calculated as the mean paired difference divided by the standard deviation of the paired differences.

Internal consistency was evaluated using Cronbach’s alpha coefficient, corrected item-total correlations, and Cronbach’s alpha if item deleted. The combined CRSwNP patient sample was treated as the primary population for internal consistency estimation; subgroup and pooled all-participant analyses were secondary. Internal consistency analyses were performed using complete-case data; participants with missing item-level responses were excluded from these analyses.

Exploratory associations relevant to construct validity were examined using Spearman’s rank correlation coefficient between SNOT-22 total scores and Lund–Kennedy endoscopic scores. These analyses were not considered sufficient to establish convergent validity.

All statistical tests were two-sided, and *p* values < 0.05 were considered statistically significant.

Artificial intelligence (AI)-assisted language tools were used solely to improve the clarity, grammar, and readability of the English language in this manuscript. AI was not used for the study design, data collection, data analysis, interpretation of results, or generation of scientific content. All scientific conclusions, analyses, and final manuscript content were reviewed and approved by the authors, who take full responsibility for the accuracy and integrity of the work.

## 3. Results

### 3.1. Participant Characteristics

A total of 151 participants were enrolled, including 50 healthy volunteers, 50 patients in the vitamin D supplementation group, and 51 patients in the standard treatment group. Baseline demographic and clinical characteristics are summarized in Table 1.

### 3.2. Internal Consistency

Internal consistency was assessed using item-level responses from the Kazakh-language SNOT-22. Complete questionnaires were available for 149 participants overall and for 99 patients with CRSwNP. Two patient questionnaires (one from the vitamin D supplementation group and one from the standard treatment group) contained a missing response to a single item and were excluded from the complete-case internal consistency analysis. In the combined target CRSwNP sample (*n* = 99), Cronbach’s alpha was 0.921. Corrected item-total correlations ranged from 0.374 to 0.767, and Cronbach’s alpha if item deleted ranged from 0.913 to 0.921. Subgroup coefficients were 0.901 in the vitamin D supplementation group (*n* = 49) and 0.934 in the standard treatment group (*n* = 50); the healthy volunteer coefficient was 0.891 (*n* = 50). The pooled all-participant coefficient was 0.945 and is reported only as a secondary descriptive estimate because combining clinically distinct groups broadens score variability and may influence alpha. All estimates should be interpreted cautiously because structural validity was not evaluated. All these internal consistency metrics and detailed subgroup coefficients are comprehensively summarized in Table 2.

**Table 1:** Baseline demographic and clinical characteristics of the study population. The study groups differed significantly with respect to age and sex distribution. Patients in the standard treatment group were older than those in the vitamin D supplementation group and healthy volunteers (*p* = 0.005), and the proportion of female participants differed significantly among groups (*p* < 0.001). Baseline SNOT-22 scores differed significantly among the study groups (*p* < 0.001), with substantially lower scores observed in healthy volunteers than in patients with CRSwNP. The distribution of baseline SNOT-22 scores is shown in Figure 2.

Variable	Healthy Volunteers (*n* = 50)	Vitamin D Supplementation Group (*n* = 50)	Standard-Treatment Group (*n* = 51)	*p*-Value
Age, years, mean ± SD; median [IQR]	38.12 ± 17.71; 34 [19.5–54]	42.72 ± 14.17; 41 [34–53.75]	48.78 ± 15.16; 50 [35–63.5]	0.005
Female, *n* (%)	44 (88.0)	36 (72.0)	22 (43.1)	<0.001
Male, *n* (%)	6 (12.0)	14 (28.0)	29 (56.9)	
Baseline SNOT-22 score, mean ± SD; median [IQR]	9.68 ± 13.42; 2 [0–16.25]	47.72 ± 20.72; 47.5 [32.25–62.5]	43.80 ± 23.84; 43 [25.5–60]	<0.001
Baseline Lund-Kennedy score, mean ± SD; median [IQR]	—	10.24 ± 1.44; 10 [10–10]	11.57 ± 3.11; 10 [10–12]	0.001

Continuous variables are presented as the mean ± SD and, for non-normally distributed variables, median [IQR]; categorical variables as *n* (%). Age and baseline SNOT-22 scores were compared using the Kruskal–Wallis test; sex distribution using Pearson’s Chi-square test; and baseline Lund–Kennedy scores using the Mann–Whitney U test. All tests were two-sided.

**Table 2:** Internal consistency statistics for the Kazakh-language SNOT-22.

Sample	Participants Included in Complete-Case Analysis	Cronbach’s α	Corrected Item-Total Correlation, Range	Cronbach’s α If Item Deleted, Range	Interpretation
All CRSwNP patients	99	0.921	0.374–0.767	0.913–0.921	Primary target-population estimate; interpret cautiously without structural validity
Vitamin D supplementation group	49	0.901	0.236–0.722	0.891–0.903	High alpha; interpret cautiously without structural validity
Standard-treatment group	50	0.934	0.369–0.797	0.928–0.936	High alpha; interpret cautiously without structural validity
Healthy volunteers	50	0.891	0.145–0.755	0.878–0.893	High alpha; interpret cautiously without structural validity
All participants	149	0.945	0.443–0.778	0.940–0.945	Secondary descriptive estimate; interpret cautiously without structural validity

In the combined CRSwNP sample, corrected item-total correlations ranged from 0.374 to 0.767 and Cronbach’s alpha if item deleted ranged from 0.913 to 0.921. In subgroup analyses, corrected item-total correlations ranged from 0.236 to 0.722 in the vitamin D supplementation group, from 0.369 to 0.797 in the standard treatment group, and from 0.145 to 0.755 among healthy volunteers. These findings indicate high item interrelatedness in the target patient sample, while the pooled all-participant alpha should be regarded as a secondary descriptive estimate. None of the alpha coefficients establishes structural validity.

### 3.3. Responsiveness

Responsiveness was assessed by comparing baseline and one-month SNOT-22 scores within each clinical group, with the detailed results summarized in Table 3. Changes over time were analyzed using the Wilcoxon signed-rank test. Cohen’s d_z_ was retained as a standardized descriptive index of change magnitude; statistical significance and effect size were not interpreted as evidence of treatment efficacy or as proof of clinically important change. No minimal clinically important difference (MCID) or patient-reported external anchor was evaluated in this study.

In the vitamin D supplementation group, the mean SNOT-22 score decreased from 47.72 ± 20.72 at baseline to 16.32 ± 13.60 after one month (*p* < 0.001). The corresponding Cohen’s d_z_ was 1.75, indicating a large magnitude of change. In the standard treatment group, the mean SNOT-22 score decreased from 43.80 ± 23.84 to 19.84 ± 15.66 (*p* < 0.001), with a Cohen’s d_z_ of 1.13.

Both SNOT-22 and Lund–Kennedy scores changed substantially over the one-month follow-up period. These findings support the ability of the Kazakh-language SNOT-22 to detect longitudinal score change. They should not be interpreted as evidence of comparative treatment efficacy or as proof of clinically important change because no MCID or patient-reported external anchor was evaluated.

The magnitude of within-group change in SNOT-22 scores is illustrated in Figure 3. Both clinical groups demonstrated marked reductions in symptom scores after one month. No between-group treatment effect conclusion was drawn from these descriptive changes.

### 3.4. Exploratory Association with Endoscopic Findings

Exploratory associations between SNOT-22 total scores and Lund–Kennedy endoscopic scores were examined at baseline and one month, with the detailed correlation coefficients presented in Table 4. These analyses were not treated as definitive evidence of convergent validity.

At baseline, very weak and non-significant associations were observed between patient-reported symptom burden and endoscopic disease severity. In the vitamin D supplementation group, Spearman’s correlation coefficient was 0.125 (*p* = 0.388), whereas in the standard treatment group it was 0.093 (*p* = 0.516).

At the one-month follow-up, statistically significant positive correlations were identified in both groups. In the vitamin D supplementation group, a very strong correlation was observed between SNOT-22 and Lund–Kennedy scores (Spearman’s correlation coefficient = 0.826, *p* < 0.001). In the standard treatment group, the association was strong (Spearman’s correlation coefficient = 0.601, *p* < 0.001).

Correlations were very weak and non-significant at baseline but stronger after treatment. Because the association was inconsistent across time points and no prespecified convergent validity hypothesis or independent patient-reported comparator was used, these findings provide only limited exploratory evidence regarding construct validity.

Figure 4 illustrates the relationship between SNOT-22 and Lund–Kennedy scores at baseline and one month after treatment in the vitamin D supplementation and standard-treatment groups.

## 4. Discussion

This study presents an initial psychometric evaluation of the Kazakh-language SNOT-22 in patients with CRSwNP. The results show high internal consistency, clear score separation between patients and healthy volunteers, and substantial longitudinal score changes after treatment. However, these findings do not constitute a complete psychometric validation. Test–retest reliability, measurement error, and structural validity were not assessed; the known-groups comparison was affected by demographic imbalance; and associations with Lund–Kennedy scores provided only limited exploratory evidence regarding construct validity.

The principal findings for known-group comparison, longitudinal change, and exploratory endoscopic associations are illustrated in Figure 2, Figure 3 and Figure 4.

### 4.1. Internal Consistency

In the combined target CRSwNP sample with complete item-level data (*n* = 99), Cronbach’s alpha was 0.921, with corrected item-total correlations ranging from 0.374 to 0.767. This combined patient estimate is considered the primary internal consistency result because CRSwNP patients constitute the target population. Subgroup alpha coefficients were 0.901 and 0.934, while the healthy volunteer coefficient was 0.891. The pooled all-participant coefficient (0.945) is retained only as a secondary descriptive estimate because combining healthy volunteers and patients increases between-person score variability and may influence alpha. These coefficients provide evidence of internal consistency but do not establish test-retest reliability or structural validity.

Internal consistency should not be interpreted as evidence of structural validity. The SNOT-22 includes items covering sinonasal symptoms as well as sleep-related, functional, and emotional complaints. Accordingly, a high Cronbach’s alpha indicates substantial interrelatedness among questionnaire items but does not establish the dimensional structure of the Kazakh-language version. Because structural validity was not assessed in the present study, no conclusions can be drawn regarding the dimensional or factor structure of the instrument [10,25,30].

Corrected item-total correlations in the combined CRSwNP sample ranged from 0.374 to 0.767, and alpha-if-item-deleted values ranged from 0.913 to 0.921. These results are compatible with substantial item–total association in the target patient population, but their interpretation remains conditional on the untested dimensional structure of the Kazakh version.

Lower item-total correlations were observed in some subgroup analyses, particularly among healthy volunteers, where restricted score variability and floor effects are plausible. Accordingly, subgroup item-level results should be interpreted cautiously; the combined CRSwNP sample remains the primary basis for interpreting internal consistency [11,22,31].

### 4.2. Context of Previous Language Adaptations

Selected previous SNOT-22 language adaptations are summarized in Table 5 to provide methodological context. Direct numerical comparison should be interpreted cautiously because populations, sample sizes, retest intervals, and analytic approaches differed across studies. Several prior adaptations included short-interval test–retest assessment, whereas this property was not evaluated in the present study.

### 4.3. Known-Group Validity

Baseline SNOT-22 scores were markedly lower in healthy volunteers than in patients with CRSwNP, providing preliminary support for the expected known-group contrast. However, this comparison cannot be regarded as an unconfounded test of known-groups validity because the groups differed significantly in age and sex distribution.

This observation is consistent with the intended purpose of SNOT-22 as a disease-specific patient-reported outcome measure. In addition to rhinologic symptoms, the questionnaire captures sleep disturbance, functional limitations, fatigue, and emotional consequences, thereby reflecting the broader impact of CRSwNP on daily functioning and health-related quality of life [7,9,31].

The demographic imbalance is methodologically important: healthy volunteers were younger and included a substantially higher proportion of women than the patient groups. Age- and sex-related differences could have contributed to SNOT-22 score variation independently of disease status. Nevertheless, the magnitude of score separation was large: the median baseline SNOT-22 score was 2 [IQR 0–16.25] in healthy volunteers compared with 47.5 [32.25–62.5] and 43 [25.5–60] in the two CRSwNP groups. Demographic imbalance may, therefore, have contributed to between-group variability but is unlikely to account fully for the observed magnitude of separation. Because no age- and sex-adjusted analysis was performed, however, the known-group findings remain preliminary rather than definitive.

Future studies should evaluate known-groups validity in age- and sex-matched populations and include patients with other sinonasal disorders to further assess the discriminative performance of the Kazakh-language SNOT-22 across different clinical settings.

### 4.4. Responsiveness

SNOT-22 scores decreased substantially from baseline to one month in both clinical groups. Wilcoxon tests and standardized effect sizes describe within-group longitudinal change and support responsiveness over this interval; they do not establish comparative treatment efficacy or demonstrate that change exceeded an MCID. In the absence of an external patient-reported anchor or an empirically established MCID for the Kazakh version, the observed changes are therefore described as substantial score changes rather than clinically meaningful changes.

It should be noted that responsiveness and test–retest reliability represent distinct measurement properties. Test-retest reliability requires repeated administration under conditions in which the underlying clinical status is expected to remain stable. In the present study, the only repeated patient assessment was performed one month after endoscopic sinus surgery and medical treatment, when clinical improvement was expected; these data therefore measure sensitivity to change rather than score stability [22,23,24,30]. Healthy volunteers were included to assess known-group validity and completed the questionnaire only once. Although a second administration by telephone would have been technically feasible, such a retest was not prospectively incorporated into the study protocol. A valid reliability assessment would require a predefined retest interval, a standardized administration procedure, and confirmation that participants remained clinically stable between measurements. We therefore regard the absence of a prospectively planned test–retest assessment as a methodological limitation rather than attempting a retrospective reliability estimate from post-treatment data.

Overall, the marked reduction in SNOT-22 scores after treatment (Figure 3) supports the suitability of the Kazakh-language questionnaire for monitoring longitudinal changes in patients with CRSwNP.

### 4.5. Association with Endoscopic Findings

At baseline, correlations between SNOT-22 and Lund–Kennedy scores were very weak and non-significant in both clinical groups. Positive correlations were observed at one month, but the inconsistency between baseline and follow-up limits the strength of the evidence.

SNOT-22 and Lund–Kennedy scores assess related but distinct aspects of CRSwNP: the former reflects patient-reported symptom burden and health-related quality of life, whereas the latter reflects endoscopic disease findings. Although an association between these measures may be expected, the anticipated direction and magnitude of the correlation should ideally be specified a priori as part of a construct validity hypothesis. Because no such hypothesis was prespecified and baseline associations were absent, the present findings should be interpreted as exploratory rather than confirmatory evidence of convergent validity [25,30].

The stronger correlations observed after treatment suggest closer alignment between subjective symptoms and objective endoscopic findings following clinical improvement. One possible explanation is that treatment reduced some of the heterogeneity present at baseline, resulting in greater concordance between patient-reported and endoscopic outcomes. However, post-treatment correlations should be interpreted cautiously because they may also be influenced by the overall direction of clinical change and the reduced variability of follow-up scores.

Taken together, the endoscopic correlation findings should be regarded as exploratory rather than as confirmation of convergent validity. The results are compatible with the complementary roles of patient-reported and endoscopic assessments in CRSwNP, but additional construct-validity testing against prespecified hypotheses and appropriate comparator PROMs is required.

### 4.6. Clinical Implications

The cross-culturally adapted Kazakh-language SNOT-22 provides a patient-reported measure for describing symptom burden and longitudinal score change in Kazakh-speaking patients with CRSwNP. The present findings support its use for research-level assessment of these selected measurement properties, but they do not establish complete psychometric validation. Until test–retest reliability, measurement error, structural validity, and an anchor-based threshold for meaningful change have been established, interpretation of individual change should remain cautious. SNOT-22 should be used as a complement to, rather than a substitute for, endoscopic and other clinical assessments [33,34,35,36].

### 4.7. Strengths, Limitations, and Future Research

A methodological strength of the study was the evaluation of several complementary measurement characteristics within the target CRSwNP population, together with a healthy reference group and longitudinal follow-up. The combined CRSwNP sample was used as the primary population for internal consistency estimation, while the healthy group provided an exploratory known-group contrast.

Several limitations constrain interpretation. The study was conducted at a single center, and healthy volunteers differed from patients in age and sex distribution; therefore, the known-group comparison should be regarded as preliminary. A small number of clinically eligible healthy volunteers had elevated SNOT-22 scores, and the protocol did not require repeat clinical reassessment solely because of a high questionnaire score. Test–retest reliability and measurement error were not assessed because no repeat administration was scheduled under clinically stable conditions. Structural validity, measurement invariance, and an anchor-based minimal clinically important difference were also not evaluated. Although the available sample supported the planned hypothesis-testing and exploratory analyses, it was not designed for a comprehensive factor-analytic evaluation [22,23,24,30].

Future studies should therefore include larger, multicenter and demographically balanced cohorts; prospectively planned test-retest assessment in clinically stable participants; factor-analytic evaluation of structural validity and measurement invariance; comparison with appropriate patient-reported outcome measures using prespecified construct-validity hypotheses; and anchor-based estimation of meaningful change. Longer follow-up would also help characterize responsiveness across surgical, medical, and biologic treatment contexts [33,34,37].

## 5. Conclusions

The Kazakh-language SNOT-22 showed high internal consistency, a marked patient-versus-healthy score contrast, and substantial within-group score changes over one month. These findings provide promising initial evidence for selected measurement properties, but they should be interpreted within the methodological limitations of the study.

The evidence for convergent validity was limited because baseline correlations with Lund–Kennedy scores were weak and non-significant, and the known-group comparison was potentially confounded by age and sex imbalance. High Cronbach’s alpha should also be interpreted cautiously in the absence of structural validity.

Before the Kazakh-language SNOT-22 can be considered fully validated, further studies should assess test–retest reliability, measurement error, structural validity, measurement invariance, and an anchor-based threshold for meaningful change in larger and demographically balanced samples.

## Figures and Tables

**Figure 1:**
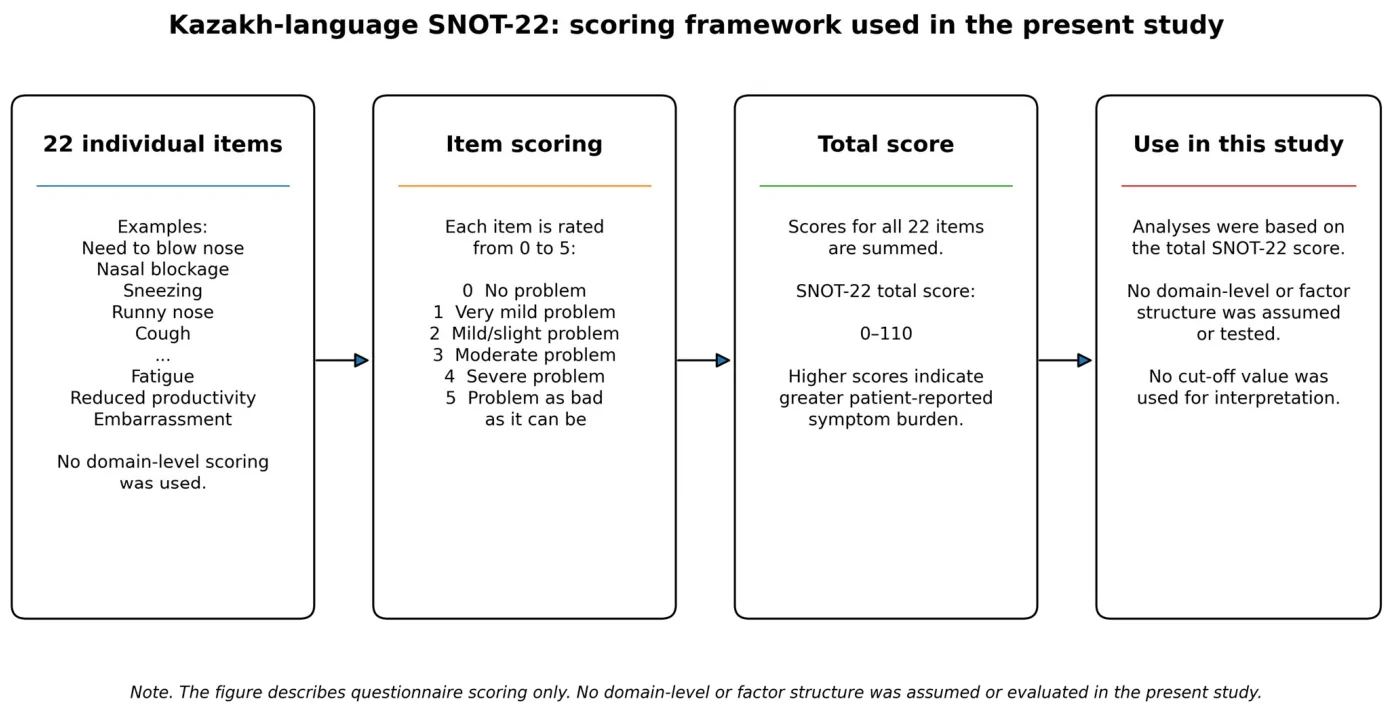
Scoring framework of the Kazakh-language SNOT-22 used in the present study. The figure illustrates item-level scoring and calculation of the total score only. No domain-level or factor structure was assumed or tested.

**Figure 2:**
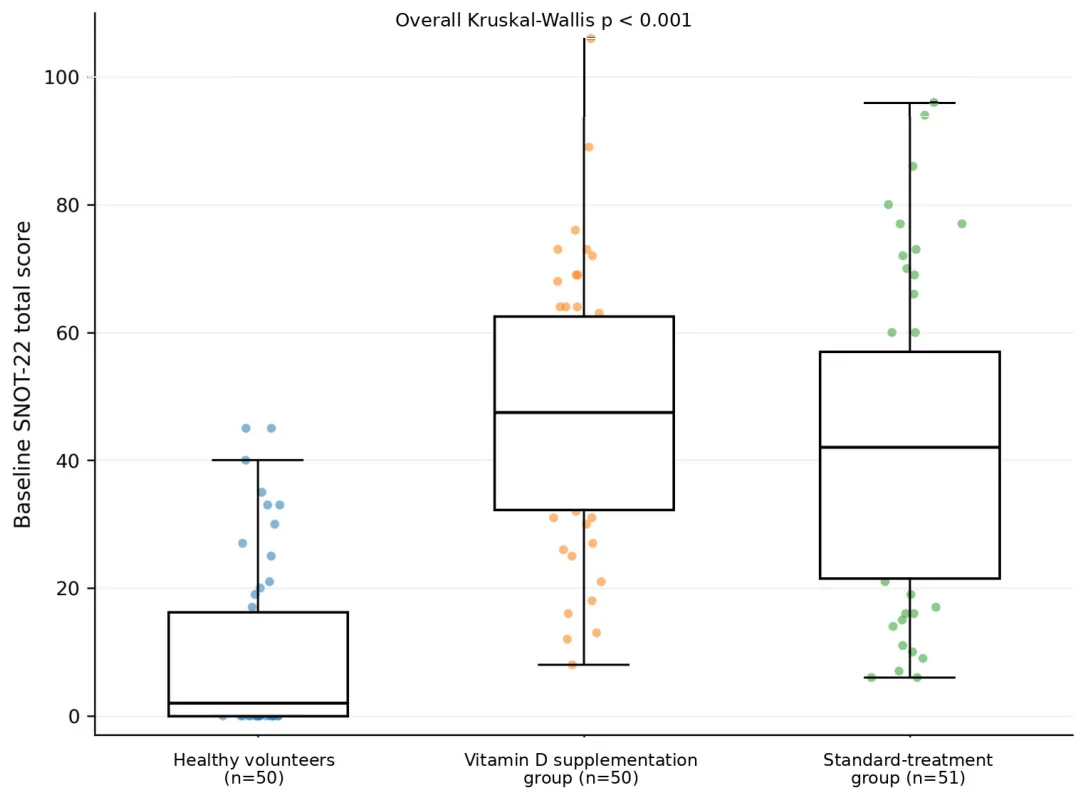
Distribution of baseline SNOT-22 scores in healthy volunteers and patients with CRSwNP. Boxes indicate the interquartile range, horizontal lines represent the median, whiskers indicate 1.5 × IQR, and dots represent individual observations. Overall Kruskal–Wallis test: *p* < 0.001; no pairwise post hoc comparisons are displayed. Most healthy volunteers had low SNOT-22 scores, although a small number showed higher values. Control eligibility was based on prespecified clinical screening criteria, including the absence of sinonasal symptoms and a normal nasal endoscopic examination, rather than on a SNOT-22 score threshold. Accordingly, isolated higher questionnaire scores were retained when the clinical eligibility criteria were met. The SNOT-22 includes sleep-related, fatigue, functional, and emotional items that are not specific to chronic rhinosinusitis; therefore, an elevated total score in an otherwise eligible control participant is not necessarily indicative of occult CRS. The study protocol did not mandate repeat clinical reassessment solely on the basis of an unexpectedly high SNOT-22 score, which should be considered when interpreting the control-group distribution. Among patients with CRSwNP, baseline Lund–Kennedy scores were significantly higher in the standard-treatment group than in the vitamin D supplementation group (*p* = 0.001).

**Figure 3:**
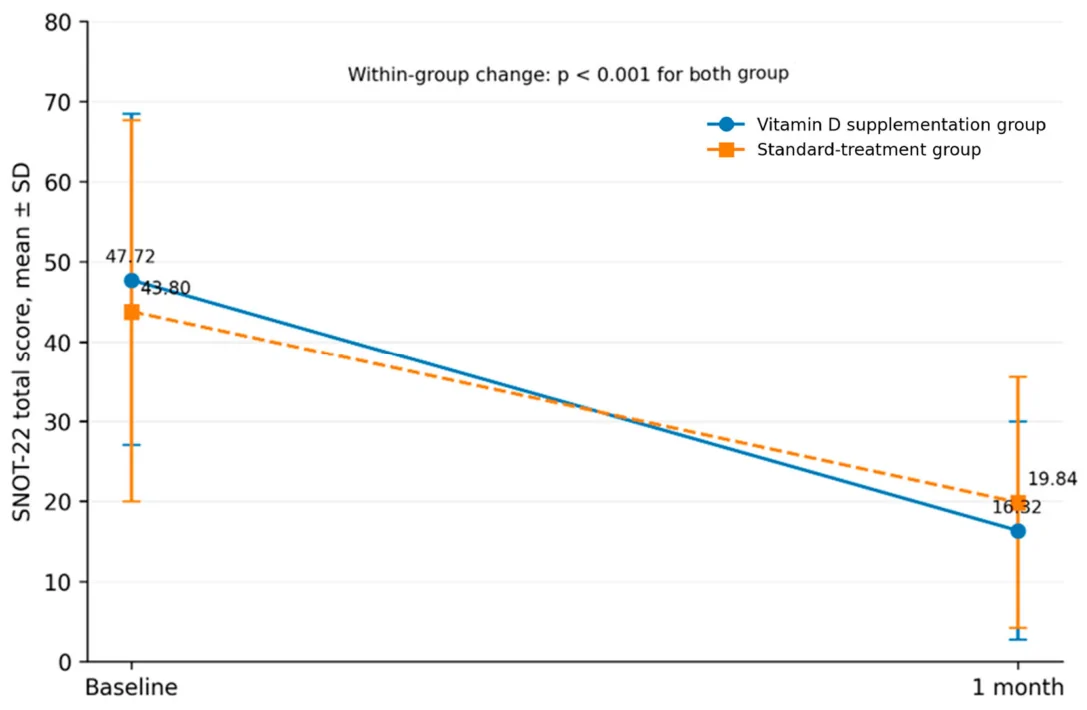
Changes in mean SNOT-22 scores from baseline to one month after treatment. Error bars represent standard deviations.

**Figure 4:**
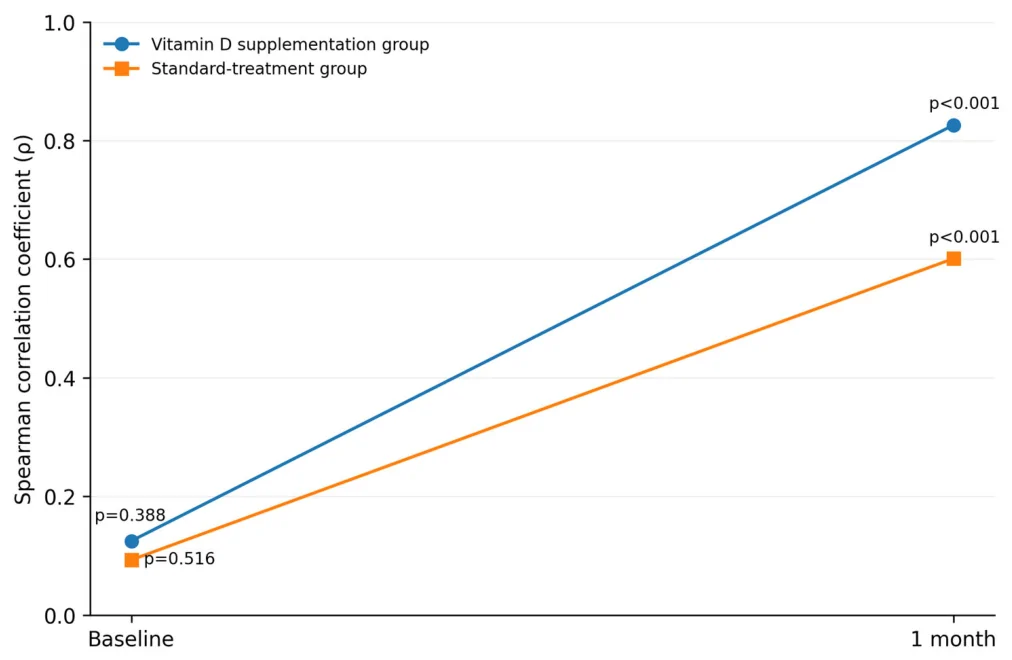
Spearman correlations between SNOT-22 and Lund–Kennedy scores.

**Table 3:** Longitudinal changes in SNOT-22 and Lund–Kennedy scores after one month of follow-up.

Outcome	Vitamin D Supplementation Group				Standard-Treatment Group			
	Baseline	1 Month	*p* Value	Cohen’s d_z_	Baseline	1 Month	*p*-Value	Cohen’s d_z_
SNOT-22 score, mean ± SD; median [IQR]	47.72 ± 20.72; 47.5 [32.25–62.5]	16.32 ± 13.60; 12 [5.5–26.25]	<0.001	1.75	43.80 ± 23.84; 43 [25.5–60]	19.84 ± 15.66; 14 [5–32]	<0.001	1.13
Lund–Kennedy score, mean ± SD; median [IQR]	10.24 ± 1.44; 10 [10–10]	3.76 ± 2.31; 2 [2–6]	<0.001	2.52	11.57 ± 3.11; 10 [10–12]	4.41 ± 2.79; 4 [2–6]	<0.001	1.86

Note: Values are presented as the mean ± SD and median [IQR]. Within-group changes were analyzed using the Wilcoxon signed-rank test. Cohen’s d_z_ is a standardized descriptive index of change and is not interpreted as treatment efficacy or as an MCID.

**Table 4:** Exploratory associations between SNOT-22 and Lund–Kennedy scores.

Time Point	Vitamin D Supplementation Group, Spearman’s Rho	*p*-Value	Interpretation	Standard Treatment Group, Spearman’s Rho	*p*-Value	Interpretation
Baseline	0.125	0.388	Very weak; not significant	0.093	0.516	Very weak; not significant
1 month	0.826	<0.001	Very strong positive association	0.601	<0.001	Strong positive association

Spearman’s rank correlation was used because the score distributions were non-normal/ordinal. Correlation strength was described as very weak (<0.20), weak (0.20–0.39), moderate (0.40–0.59), strong (0.60–0.79), or very strong (≥0.80). All tests were two-sided.

**Table 5:** Selected measurement properties reported for different language versions of the SNOT-22.

Language/Country	Year	Sample Size (*n*)	Cronbach’s α	Test–Retest Reliability	Responsiveness
Original English (Hopkins et al.) [32]	2009	2803	0.91	Evaluated	Effect size 0.81; MID 8.9
Arabic (Saudi Arabia) [12]	2018	119	0.94	ICC = 0.99	Demonstrated
Polish [14]	2024	148	0.89	ICC = 0.977	Not evaluated
Hindi [15]	2022	110	0.823–0.868	Evaluated	Hedges’ g = 2.34
Kazakh (present study)	2026	99 CRSwNP complete cases	0.921	Not evaluated	Substantial score change at 1 month

Note: The table includes selected representative language versions for descriptive context only. Terminology and analytic methods differed between studies, and values should not be interpreted as demonstrating psychometric equivalence. For the Kazakh version, Cronbach’s alpha is reported for the combined CRSwNP complete-case sample (*n* = 99), which was treated as the primary target population. Test-retest reliability and structural validity remain to be established.

## Data Availability

Data available in a publicly accessible repository. The data presented in this study are openly available in 10.6084/m9.figshare.33160628.

## Abbreviations

The following abbreviations are used in this manuscript:

CRS	Chronic rhinosinusitis
CRSwNP	Chronic rhinosinusitis with nasal polyps
EPOS 2020	The European Position Paper on Rhinosinusitis and Nasal Polyps
SNOT-22	The Sino-Nasal Outcome Test-22
